# Editorial: Crop Traits for Defense against Pests and Disease: Durability, Breakdown and Future Prospects

**DOI:** 10.3389/fpls.2017.00209

**Published:** 2017-02-21

**Authors:** Scott N. Johnson, Alison J. Karley, Peter J. Gregory, Rex M. Brennan

**Affiliations:** ^1^Hawkesbury Institute for the Environment, Western Sydney UniversityPenrith, NSW, Australia; ^2^The James Hutton InstituteDundee, UK; ^3^School of Agriculture, Policy and Development, University of ReadingReading, UK

**Keywords:** climate change, crops, food security, pathogens, pests, resistance

With the Earth's population expected to reach 11.2 billion by 2100 there is a pressing need to maximize food production at a time when productivity of many crops is reaching a plateau. Minimizing losses to pests and diseases is therefore a crucial means of meeting this challenge and securing food supply. This research topic addresses some of the most important issues in the future sustainability of global crop production. The topic consists of 20 papers, of which 13 describe original research. There are six review papers, plus one hypothesis and theory contribution.

The development of plants with resistance to the most damaging pests and diseases is increasingly important in the face of growing pressure to reduce synthetic chemical inputs, including pesticides, fungicides, and herbicides used for crop protection. This reduction is partly underwritten by legislative directives, consumer demands and indeed an overall reduction in available chemical controls for most crops (Hillocks, 2012), especially those outwith the main arable species. Additionally, losses due to pest and disease attacks represent a major financial cost in crop production and throughout the subsequent supply chain, even before the costs of control measures are considered. The move toward more sustainable production systems, based on integrated pest and disease management approaches, is gaining momentum, and the development of more resistant cultivars is a major factor contributing to the success of these systems. In addition, there is a recognized need for frameworks (e.g., Birch et al., 2011) which combine natural enemies with resistant cultivars and other management practices to reduce reliance on crop protection chemicals and maintain viable and sustainable future crop production.

The development of resistant cultivars is crucial to the future of sustainable crop production practices, and there is a substantial need for the continued introgression of specific resistance genes or physical and structural traits, from existing or extended genetic resources. Recent advances in knowledge can aid this process, even in many minor crop species, where increased understanding of trait heritability and technological advances in genomics and bioinformatics are enabling the identification of genes controlling resistance, providing a framework for improved selection efficiency. Moreover, the advent of new technologies can provide significant benefits, as exemplified by the opportunities afforded by CRISPR-based tools in understanding plant-pathogen interactions; these are addressed in the review by Barakate and Stephens. The need for new resistance genes is crucial within many pathogen/crop systems, and Van Weymers et al. describe the application of a range of “omics” technologies in potato to identify novel resistances to potato blight within a large germplasm collection. This approach is one that can be adapted for other species and pathogens, as the future role of resistance genes from existing but underexploited genetic resources is likely to be significant. This is not limited to inherent crop resistance, however, and Reynolds et al. also highlight the role of “omics” approaches for augmenting plant resistance to invertebrates using application of silicon.

Breeding strategies focused on the identification and incorporation of specific resistance genes, both individually or in effective groups, are being developed and implemented in many active breeding programmes. The use of marker-assisted backcross breeding in rice to pyramid resistance genes for bacterial blight and blast diseases is the subject of the paper by Abhilash Kumar et al. An investigation of genes involved in the infection processes by *Phytophthora capsici* in *Capsicum* spp. by Zhang et al. through genome-wide identification highlights the role of SQUAMOSA promoter binding protein (SBP)-box genes that can be utilized in future breeding and development research. Genetic mapping of host plants, such as *Capsicum*, was used by Barbary et al. to identify QTLs linked to resistance to *Meloidogyne* nematode species, and from this the underlying genes can eventually be identified and utilized in the breeding of resistant plants. Similarly, wild *Vitis* genotypes studied by Wan et al. to find sources of resistance to *Botrytis*, based on antioxidant activity linked to resistance, will be deployed in future breeding strategies. Further work on wild *Vitis* by Wen et al. identified a gene linked to powdery mildew resistance and confirmed its effect through ectopic expression in *Arabidopsis*; again, this provides a resource for future breeding.

The effects of pest attacks on host plant metabolism was investigated by Liu et al., using the wild brassica species *Barbarea vulgaris* and the global pest diamondback moth, *Plutella xylostella*. By examining changes in glucosinolate biosynthesis induced by larval infestation, inferences can be made about the mechanisms underpinning defense induction. A quantitative approach to the breeding of resistant cultivars is described by Mohammed et al. in *Sorghum*, where the use of diallel progeny enabled the identification of a significant interaction between resistance to shoot fly and morphological traits such as grain yield and seed size. The information on the quantitative genetics of *Sorghum* can be used to inform decisions on parental choice for resistance breeding.

Many host-pathogen systems are subject to rapid evolution, for example in pathogen race structure or breakdown of host resistance. The paper by Gómez-Cortecero et al. describes the use of SNPs and SSRs to study diversity in global populations of *Neonectria ditissima* affecting apple trees, and also present evidence of a relatively simple pattern of host response which is not influenced by any race structure in the pathogen population.

Integrated Pest and Disease Management (IPDM) approaches to pest and disease control require the development of resistant varieties and also monitoring and management strategies that can be applied effectively within crops. The paper by Joshi et al. deals with the development of a susceptibility index for codling moth on apple, based on oviposition preferences. The review by Peterson et al. considers the wide range of plant defense traits that can influence the responses of natural enemies used in IPDM systems, and also includes the potential impacts of transgenic crops on trophic levels and arthropod communities.

Plant defenses can be exploited to enhance resistance to pest attack and also to confer tolerance of pest infestations. Plant physical defenses can offer particularly durable resistance to pests and pathogens (Johnson et al., 2016; Moore and Johnson, 2017); ecological studies, for example, have shown plant physical traits to be more effective deterrents to insect herbivory than plant secondary metabolites (Peeters et al., 2007; Cooke and Leishman, 2012). As outlined in the review by Mitchell et al., plant structural traits such as trichomes, spines, and cuticles can provide a physical barrier to arthropod pest attachment, feeding and oviposition, while plant vigor and altered phenology can increase tolerance of pest damage and reduce the incidence of pest attacks. This paper highlights new avenues for discovery of plant defensive traits, particularly through research to understand pest-induced changes in plant chemistry and mechanisms of plant defense “priming”.

The effects of a changing climate are likely to alter both severity of pest and pathogen attacks, and also the spectrum of organisms that will cause damage to crop plants (Johnson and Jones, 2017). Two papers in this research topic consider changes under conditions of elevated CO_2_; the first by McKenzie et al. presents data illustrating the changes in herbivory by two pests of raspberry, the European large raspberry aphid (*Amophorophoa idaei*) and the root-feeding vine weevil (*Otiorhynchus sulcatus*). The second contribution, a review by Sun et al., examines changes in host plant metabolism and water-use efficiency instigated under elevated CO_2_ conditions, and how these changes impact on the outcome of plant-aphid interactions.

Host plant resistance can have significant effects on organisms and trophic levels beyond the target pest or pathogen, and this has implications for integrated management of crop systems. This area is discussed by Peterson et al. in their review, which also considers the use of transgenic crops. Genetic modification technology has potential application in the development of pest-resistant cultivars of some arable species, especially where there are limited sources of resistance within the genus, and this is discussed in the context of cotton production systems by Trapero et al. and de Oliveira et al. The identification by the latter authors of resistances covering multiple pests may offer particular opportunities.

Making use of multiple resistance traits might also offer an alternative approach toward pathogen control. The theory paper by Newton suggests that the harmful effects of fungal disease outbreaks in cereal crops could be limited by using mixed genotype plantings; cultivar mixtures often show higher tolerance of, or resistance to, disease. The impact of this approach on other microbial species in the crop environment is also considered.

The development of environmentally friendly control mechanisms for pests is a further aspect of future crop production systems, and there are various less damaging ingredients under consideration. These can include the use of plant mutualists or manipulation of soil conditions, for example by application of chemical constituents such as silicon (Johnson et al., 2016). In terms of plant mutualists, arbuscular mycorrhizal fungi can improve crop productivity, as reported by Robinson-Boyer et al., which may result in better tolerance of pest and disease attack Mitchell et al. and also help plants resist attack by root herbivores (Johnson et al., 2016). Moreover, the role of silicon application to the soil and resistance to herbivory is considered in the review by Reynolds et al.. They present evidence for direct and indirect defenses (e.g., recruitment of natural enemies of pests via volatile emissions) by silicon.

We hope this research topic will provide a valuable resource for workers and researchers in the field of crop protection by providing a source of information about existing and novel sources of crop resistance and tolerance traits, their incorporation into breeding programmes, how they can be deployed for maximum efficacy under field conditions when integrated with other pest and disease control measures, and their potential to provide durable and sustainable crop protection under a changing environment.

## Tribute to alain palloix

It is with sadness that we note the death of Alain Palloix, whose manuscript on resistance to root-knot nematodes Barbary et al. is included in this research topic. Alain's work over many years made a significant contribution to the understanding of pepper-pathogen interactions and resistance breeding, and we extend our sympathy to his family and colleagues.

## Author contributions

All authors listed have made substantial, direct and intellectual contribution to the work and approved it for publication.

### Conflict of interest statement

The authors declare that the research was conducted in the absence of any commercial or financial relationships that could be construed as a potential conflict of interest.
